# (2*E*)-3-(6-Chloro-2-meth­oxy­quinolin-3-yl)-1-(2,4-di­methyl­quinolin-3-yl)prop-2-en-1-one

**DOI:** 10.1107/S1600536813019399

**Published:** 2013-07-20

**Authors:** R. Prasath, S. Sarveswari, Seik Weng Ng, Edward R. T. Tiekink

**Affiliations:** a Department of Chemistry, BITS, Pilani – K. K. Birla Goa Campus, Goa 403 726, India; b Organic Chemistry Division, School of Advanced Sciences, VIT University, Vellore 632 014, India; c Department of Chemistry, University of Malaya, 50603 Kuala Lumpur, Malaysia; d Chemistry Department, Faculty of Science, King Abdulaziz University, PO Box 80203 Jeddah, Saudi Arabia

## Abstract

The mol­ecule of the title compound, C_24_H_19_ClN_2_O_2_, is bent, with the dihedral angle between the terminal quinoline ring systems being 63.30 (5)°. The quinolinyl residues are connected by an almost planar prop-2-en-1-one bridge (r.m.s. deviation = 0.022 Å), with the dihedral angles between this plane and the appended quinolinyl residues being 75.86 (7) and 38.54 (7)°. The C atom of the meth­oxy group is close to coplanar with its attached ring [deviation = 0.116 (2) Å]. In the crystal, a three-dimensional architecture is constructed by meth­yl–carbonyl C—H⋯O inter­actions and π–π inter­actions between centrosymmetrically related quinolinyl residues [centroid-to-centroid separations 3.5341 (10) and 3.8719 (9) Å].

## Related literature

For background to the biological activities and photophysical properties of quinolines, and their utility as inter­mediates in organic synthesis, see: Prasath & Bhavana (2012 ▶); Joshi *et al.* (2011 ▶). For background to the bio-activities of quinolinyl chalcones, see: Prasath *et al.* (2013*a* ▶). For a related structure, see: Prasath *et al.* (2013*b* ▶).
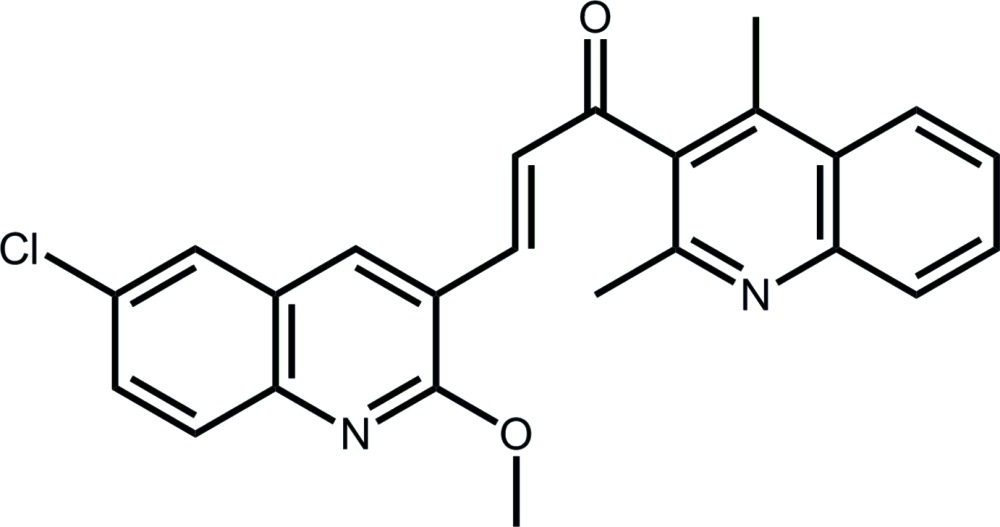


## Experimental

### 

#### Crystal data

C_24_H_19_ClN_2_O_2_
*M* *_r_* = 402.86
Monoclinic, 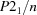

*a* = 13.1605 (3) Å
*b* = 10.4876 (2) Å
*c* = 14.8786 (3) Å
β = 106.354 (2)°
*V* = 1970.49 (7) Å^3^
*Z* = 4
Cu *K*α radiation
μ = 1.90 mm^−1^
*T* = 100 K
0.35 × 0.30 × 0.25 mm

#### Data collection

Agilent SuperNova Dual diffractometer with Atlas detector
Absorption correction: multi-scan (*CrysAlis PRO*; Agilent, 2013 ▶) *T*_min_ = 0.711, *T*_max_ = 1.000
8189 measured reflections
4053 independent reflections
3580 reflections with *I* > 2σ(*I*)
*R*_int_ = 0.019

#### Refinement

*R*[*F* ^2^ > 2σ(*F* ^2^)] = 0.044
*wR*(*F* ^2^) = 0.127
*S* = 1.07
4053 reflections
264 parameters
H-atom parameters constrained
Δρ_max_ = 0.61 e Å^−3^
Δρ_min_ = −0.49 e Å^−3^

### 

Data collection: *CrysAlis PRO* (Agilent, 2013 ▶); cell refinement: *CrysAlis PRO*; data reduction: *CrysAlis PRO*; program(s) used to solve structure: *SHELXS97* (Sheldrick, 2008 ▶); program(s) used to refine structure: *SHELXL97* (Sheldrick, 2008 ▶); molecular graphics: *ORTEP-3 for Windows* (Farrugia, 2012 ▶) and *DIAMOND* (Brandenburg, 2006 ▶); software used to prepare material for publication: *publCIF* (Westrip, 2010 ▶).

## Supplementary Material

Crystal structure: contains datablock(s) global, I. DOI: 10.1107/S1600536813019399/hb7107sup1.cif

Structure factors: contains datablock(s) I. DOI: 10.1107/S1600536813019399/hb7107Isup2.hkl

Supplementary material file. DOI: 10.1107/S1600536813019399/hb7107Isup3.cml

Additional supplementary materials:  crystallographic information; 3D view; checkCIF report

## Figures and Tables

**Table 1:** Hydrogen-bond geometry (Å, °)

*D*—H⋯*A*	*D*—H	H⋯*A*	*D*⋯*A*	*D*—H⋯*A*
C10—H10*B*⋯O1^i^	0.98	2.52	3.221 (2)	129

Symmetry code: (i) 
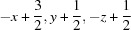
.

**Fig. 1.**
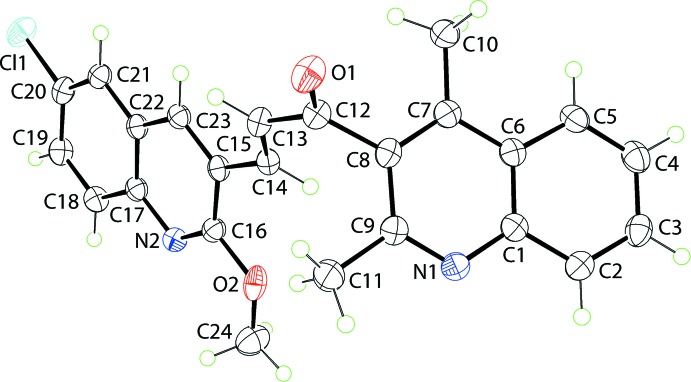
The molecular structure of (I), showing displacement ellipsoids at the 50% probability level.

**Fig. 2.**
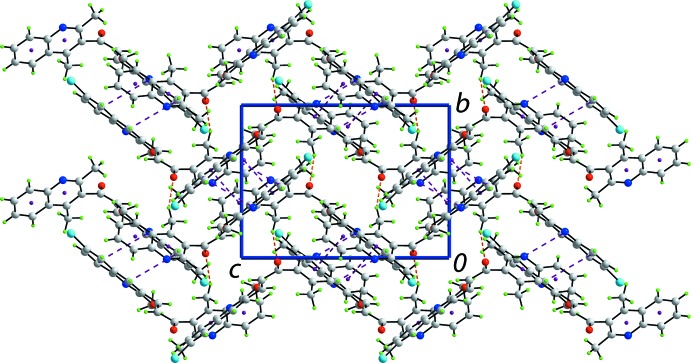
A view, in projection down the a axis, of the unit-cell contents of (I). The C—H···O and π–π interactions are shown as orange and purple blue dashed lines, respectively.

## supplementary crystallographic information

### Comment

Quinoline derivatives are an important class of natural and synthetic products, which possess a number of interesting biological activities, are valuable intermediates in organic synthesis, and exhibit a multitude of photo-physical properties (Prasath & Bhavana, 2012; Joshi *et al.*, 2011). Also, quinolinyl chalcones have gained much attention due to their bio-activity, such as anti-bacterial, anti-fungal, anti-malarial and anti-cancer activities (Prasath *et al.*, 2013*a*). It was in this connection that the title compound, (I), was investigated.

The molecular structure of (I), Fig. 1, comprises two quinolinyl rings connected by the ends of a prop-2-en-1-one bridge. The dihedral angle between the quinolinyl rings is 63.30 (5)°. The methoxy group is coplanar with the quinolinyl ring to which it is attached, as seen in the value of the C24—O2—C16—N2 torsion angle of 3.1 (2)°. The conformation about the ethylene bond [C13═C14 = 1.335 (2) Å] is *E*. The central C_5_O plane comprising the O1, C8 and C12–C15 atoms, is almost planar, with an r.m.s. deviation of 0.022 Å. The N1- and N2-containing quinolinyl rings form dihedral angles of 75.86 (7) and 38.54 (7)°, respectively, with the central plane.

In the most closely related structure, (II), namely (2*E*)-3-(2-chloro-8-methylquinolin-3-yl)-1-(5,7-dimethylquinolin-6-yl)prop-2-en-1-one (Prasath *et al.*, 2013*b*), the dihedral angle between the quinolinyl residues is 83.72 (4)°, indicating a more open configuration than that in (I). Also, when the structures are viewed normal to the ethylene bond, the pyridyl-N atoms in (I) can be described as *syn*, whereas they are closer to *anti* in (II).

In the crystal, supramolecular helical chains are formed by methyl-C—H···O(carbonyl) interactions, Table 1. These are connected into a three-dimensional architecture by π–π interactions between the rings of centrosymmetrically related N1-quinolinyl residues [3.5341 (10) Å; angle of inclination = 2.48 (9)° for symmetry operation 1 - *x*, 1 - *y*, -*z*] and between the rings of centrosymmetrically related N2-quinolinyl residues [3.8719 (9) Å; angle of inclination = 2.52 (8)° for symmetry operation 1 - *x*, 2 - *y*, -*z*], Fig. 2.

### Experimental

A mixture of 2,4-dimethyl-3-acetylquinoline (200 mg, 0.001 *M*) and 2,6-dichloroquinoline-3-carbaldehyde (230 mg, 0.001 *M*) in methanol (20 ml) containing 0.2 g of potassium hydroxide was stirred at room temperature for 12 h. At the end of the period, the reaction mixture was neutralized with dilute acetic acid and the resultant solid was filtered, dried and purified by column chromatography using ethyl acetate–hexane (2:1) mixture to afford (I). Re-crystallization was by slow evaporation of an acetone solution of (I), which yielded pale-yellow blocks in 61% yield; *M*.pt: 423–425 K.

### Refinement

Carbon-bound H atoms were placed in calculated positions [C—H = 0.95–0.98 Å, *U*_iso_(H) = 1.2–1.5*U*_eq_(C)] and were included in the refinement in the riding-model approximation.

### Crystal data

**Table:**

C_24_H_19_ClN_2_O_2_	*F*(000) = 840
*M**_r_* = 402.86	*D*_x_ = 1.358 Mg m^−^^3^
Monoclinic, *P*2_1_/*n*	Cu *K*α radiation, λ = 1.54184 Å
Hall symbol: -P 2yn	Cell parameters from 4094 reflections
*a* = 13.1605 (3) Å	θ = 3.1–76.4°
*b* = 10.4876 (2) Å	µ = 1.90 mm^−^^1^
*c* = 14.8786 (3) Å	*T* = 100 K
β = 106.354 (2)°	Block, pale-yellow
*V* = 1970.49 (7) Å^3^	0.35 × 0.30 × 0.25 mm
*Z* = 4	

### Data collection

**Table:**

Agilent SuperNova Dual diffractometer with Atlas detector	4053 independent reflections
Radiation source: SuperNova (Cu) X-ray Source	3580 reflections with *I* > 2σ(*I*)
Mirror monochromator	*R*_int_ = 0.019
Detector resolution: 10.4041 pixels mm^-1^	θ_max_ = 76.6°, θ_min_ = 4.0°
ω scans	*h* = −16→16
Absorption correction: multi-scan (*CrysAlis PRO*; Agilent, 2013)	*k* = −12→13
*T*_min_ = 0.711, *T*_max_ = 1.000	*l* = −18→18
8189 measured reflections	

### Refinement

**Table:**

Refinement on *F*^2^	Primary atom site location: structure-invariant direct methods
Least-squares matrix: full	Secondary atom site location: difference Fourier map
*R*[*F*^2^ > 2σ(*F*^2^)] = 0.044	Hydrogen site location: inferred from neighbouring sites
*wR*(*F*^2^) = 0.127	H-atom parameters constrained
*S* = 1.07	*w* = 1/[σ^2^(*F*_o_^2^) + (0.0675*P*)^2^ + 0.8422*P*] where *P* = (*F*_o_^2^ + 2*F*_c_^2^)/3
4053 reflections	(Δ/σ)_max_ = 0.001
264 parameters	Δρ_max_ = 0.61 e Å^−^^3^
0 restraints	Δρ_min_ = −0.49 e Å^−^^3^

### Special details

**Table:**

Geometry. All s.u.'s (except the s.u. in the dihedral angle between two l.s. planes) are estimated using the full covariance matrix. The cell s.u.'s are taken into account individually in the estimation of s.u.'s in distances, angles and torsion angles; correlations between s.u.'s in cell parameters are only used when they are defined by crystal symmetry. An approximate (isotropic) treatment of cell s.u.'s is used for estimating s.u.'s involving l.s. planes.
Refinement. Refinement of *F*^2^ against ALL reflections. The weighted *R*-factor *wR* and goodness of fit *S* are based on *F*^2^, conventional *R*-factors *R* are based on *F*, with *F* set to zero for negative *F*^2^. The threshold expression of *F*^2^ > 2σ(*F*^2^) is used only for calculating *R*-factors(gt) *etc*. and is not relevant to the choice of reflections for refinement. *R*-factors based on *F*^2^ are statistically about twice as large as those based on *F*, and *R*-factors based on ALL data will be even larger.

### Fractional atomic coordinates and isotropic or equivalent isotropic displacement parameters (Å^2^)

**Table:**

	*x*	*y*	*z*	*U*_iso_*/*U*_eq_	
Cl1	0.69700 (4)	1.15984 (5)	0.82007 (3)	0.03903 (15)	
O1	0.74055 (11)	0.52819 (14)	0.32665 (9)	0.0379 (3)	
O2	0.34467 (11)	0.69372 (12)	0.42578 (8)	0.0324 (3)	
N1	0.40195 (12)	0.49117 (14)	0.14020 (10)	0.0271 (3)	
N2	0.39020 (11)	0.82891 (14)	0.55617 (10)	0.0249 (3)	
C1	0.40917 (13)	0.56639 (17)	0.06701 (12)	0.0252 (3)	
C2	0.32879 (14)	0.55356 (17)	−0.01880 (12)	0.0284 (4)	
H2	0.2728	0.4945	−0.0237	0.034*	
C3	0.33138 (15)	0.62559 (19)	−0.09439 (12)	0.0319 (4)	
H3	0.2776	0.6155	−0.1519	0.038*	
C4	0.41310 (15)	0.7150 (2)	−0.08803 (13)	0.0338 (4)	
H4	0.4130	0.7664	−0.1406	0.041*	
C5	0.49274 (15)	0.72798 (18)	−0.00624 (13)	0.0302 (4)	
H5	0.5479	0.7878	−0.0026	0.036*	
C6	0.49323 (13)	0.65264 (16)	0.07298 (12)	0.0247 (3)	
C7	0.57710 (13)	0.65747 (16)	0.15858 (12)	0.0256 (4)	
C8	0.56830 (13)	0.58072 (16)	0.23097 (11)	0.0250 (3)	
C9	0.47856 (14)	0.49875 (17)	0.21924 (12)	0.0271 (4)	
C10	0.67212 (14)	0.74029 (19)	0.16571 (13)	0.0323 (4)	
H10A	0.7189	0.7374	0.2299	0.048*	
H10B	0.6491	0.8283	0.1496	0.048*	
H10C	0.7104	0.7092	0.1223	0.048*	
C11	0.46915 (18)	0.4122 (2)	0.29718 (14)	0.0394 (5)	
H11A	0.4195	0.3430	0.2712	0.059*	
H11B	0.4431	0.4610	0.3423	0.059*	
H11C	0.5388	0.3762	0.3288	0.059*	
C12	0.65569 (14)	0.57938 (17)	0.32225 (12)	0.0269 (4)	
C13	0.63785 (14)	0.64480 (17)	0.40412 (12)	0.0270 (4)	
H13	0.6935	0.6466	0.4609	0.032*	
C14	0.54619 (13)	0.70161 (16)	0.40128 (11)	0.0242 (3)	
H14	0.4909	0.6960	0.3444	0.029*	
C15	0.52396 (13)	0.77189 (16)	0.47882 (11)	0.0231 (3)	
C16	0.41937 (13)	0.76998 (16)	0.49044 (11)	0.0247 (3)	
C17	0.46563 (13)	0.90084 (16)	0.61874 (11)	0.0237 (3)	
C18	0.43667 (14)	0.96369 (17)	0.69184 (12)	0.0278 (4)	
H18	0.3674	0.9532	0.6983	0.033*	
C19	0.50854 (15)	1.04000 (17)	0.75364 (12)	0.0293 (4)	
H19	0.4891	1.0819	0.8029	0.035*	
C20	0.61116 (14)	1.05587 (17)	0.74370 (12)	0.0282 (4)	
C21	0.64352 (14)	0.99357 (16)	0.67556 (12)	0.0259 (3)	
H21	0.7135	1.0042	0.6707	0.031*	
C22	0.57067 (13)	0.91316 (15)	0.61268 (11)	0.0226 (3)	
C23	0.59830 (13)	0.84460 (15)	0.54060 (11)	0.0235 (3)	
H23	0.6684	0.8492	0.5352	0.028*	
C24	0.24358 (17)	0.6907 (2)	0.43289 (16)	0.0437 (5)	
H24A	0.2003	0.6347	0.3843	0.066*	
H24B	0.2138	0.7770	0.4247	0.066*	
H24C	0.2440	0.6583	0.4947	0.066*	

### Atomic displacement parameters (Å^2^)

**Table:**

	*U*^11^	*U*^22^	*U*^33^	*U*^12^	*U*^13^	*U*^23^
Cl1	0.0375 (3)	0.0398 (3)	0.0358 (3)	−0.00176 (19)	0.00367 (19)	−0.01474 (18)
O1	0.0302 (7)	0.0444 (8)	0.0365 (7)	0.0107 (6)	0.0051 (5)	−0.0085 (6)
O2	0.0458 (8)	0.0284 (6)	0.0201 (6)	0.0077 (6)	0.0046 (5)	−0.0081 (5)
N1	0.0278 (7)	0.0249 (7)	0.0285 (7)	−0.0001 (6)	0.0077 (6)	−0.0012 (6)
N2	0.0247 (7)	0.0242 (7)	0.0260 (7)	0.0009 (5)	0.0076 (6)	0.0004 (5)
C1	0.0258 (8)	0.0247 (8)	0.0267 (8)	0.0034 (6)	0.0101 (6)	−0.0030 (6)
C2	0.0261 (8)	0.0287 (9)	0.0300 (8)	0.0027 (7)	0.0075 (7)	−0.0039 (7)
C3	0.0277 (9)	0.0392 (10)	0.0263 (8)	0.0069 (8)	0.0037 (7)	−0.0012 (7)
C4	0.0333 (9)	0.0398 (10)	0.0297 (9)	0.0066 (8)	0.0110 (7)	0.0076 (8)
C5	0.0288 (9)	0.0330 (9)	0.0315 (9)	0.0008 (7)	0.0127 (7)	0.0033 (7)
C6	0.0262 (8)	0.0242 (8)	0.0256 (8)	0.0043 (6)	0.0102 (7)	−0.0016 (6)
C7	0.0258 (8)	0.0258 (8)	0.0269 (8)	0.0028 (7)	0.0104 (7)	−0.0041 (6)
C8	0.0268 (8)	0.0244 (8)	0.0239 (8)	0.0045 (7)	0.0070 (6)	−0.0046 (6)
C9	0.0297 (9)	0.0240 (8)	0.0272 (8)	0.0016 (7)	0.0074 (7)	−0.0012 (6)
C10	0.0298 (9)	0.0374 (10)	0.0311 (9)	−0.0024 (8)	0.0109 (7)	−0.0028 (7)
C11	0.0462 (11)	0.0334 (10)	0.0354 (10)	−0.0083 (9)	0.0065 (8)	0.0062 (8)
C12	0.0267 (8)	0.0255 (8)	0.0277 (8)	0.0029 (7)	0.0064 (7)	−0.0019 (7)
C13	0.0291 (8)	0.0275 (9)	0.0222 (8)	0.0022 (7)	0.0036 (6)	−0.0009 (6)
C14	0.0275 (8)	0.0240 (8)	0.0209 (7)	0.0005 (6)	0.0066 (6)	0.0008 (6)
C15	0.0263 (8)	0.0221 (8)	0.0206 (7)	0.0032 (6)	0.0061 (6)	0.0033 (6)
C16	0.0252 (8)	0.0226 (8)	0.0248 (8)	0.0004 (6)	0.0048 (6)	0.0014 (6)
C17	0.0253 (8)	0.0222 (8)	0.0235 (7)	0.0033 (6)	0.0067 (6)	0.0035 (6)
C18	0.0283 (8)	0.0278 (8)	0.0288 (8)	0.0044 (7)	0.0107 (7)	0.0007 (7)
C19	0.0332 (9)	0.0292 (9)	0.0263 (8)	0.0061 (7)	0.0097 (7)	−0.0025 (7)
C20	0.0315 (9)	0.0253 (8)	0.0248 (8)	0.0030 (7)	0.0030 (7)	−0.0017 (6)
C21	0.0254 (8)	0.0250 (8)	0.0261 (8)	0.0024 (7)	0.0054 (6)	0.0015 (6)
C22	0.0252 (8)	0.0207 (7)	0.0214 (7)	0.0038 (6)	0.0055 (6)	0.0035 (6)
C23	0.0247 (8)	0.0226 (8)	0.0236 (8)	0.0024 (6)	0.0074 (6)	0.0026 (6)
C24	0.0387 (11)	0.0444 (12)	0.0467 (12)	−0.0044 (9)	0.0101 (9)	−0.0109 (10)

### Geometric parameters (Å, º)

**Table:**

Cl1—C20	1.7417 (18)	C10—H10C	0.9800
O1—C12	1.224 (2)	C11—H11A	0.9800
O2—C24	1.364 (3)	C11—H11B	0.9800
O2—C16	1.414 (2)	C11—H11C	0.9800
N1—C9	1.318 (2)	C12—C13	1.473 (2)
N1—C1	1.369 (2)	C13—C14	1.335 (2)
N2—C16	1.303 (2)	C13—H13	0.9500
N2—C17	1.379 (2)	C14—C15	1.466 (2)
C1—C6	1.412 (2)	C14—H14	0.9500
C1—C2	1.416 (2)	C15—C23	1.370 (2)
C2—C3	1.363 (3)	C15—C16	1.435 (2)
C2—H2	0.9500	C17—C18	1.413 (2)
C3—C4	1.409 (3)	C17—C22	1.416 (2)
C3—H3	0.9500	C18—C19	1.375 (3)
C4—C5	1.371 (3)	C18—H18	0.9500
C4—H4	0.9500	C19—C20	1.409 (3)
C5—C6	1.417 (2)	C19—H19	0.9500
C5—H5	0.9500	C20—C21	1.371 (2)
C6—C7	1.432 (2)	C21—C22	1.414 (2)
C7—C8	1.375 (2)	C21—H21	0.9500
C7—C10	1.502 (2)	C22—C23	1.421 (2)
C8—C9	1.431 (2)	C23—H23	0.9500
C8—C12	1.513 (2)	C24—H24A	0.9800
C9—C11	1.505 (2)	C24—H24B	0.9800
C10—H10A	0.9800	C24—H24C	0.9800
C10—H10B	0.9800		
			
C24—O2—C16	117.82 (14)	O1—C12—C13	121.03 (16)
C9—N1—C1	117.89 (15)	O1—C12—C8	120.25 (15)
C16—N2—C17	117.27 (15)	C13—C12—C8	118.69 (14)
N1—C1—C6	123.19 (15)	C14—C13—C12	122.30 (16)
N1—C1—C2	117.43 (16)	C14—C13—H13	118.8
C6—C1—C2	119.37 (16)	C12—C13—H13	118.8
C3—C2—C1	120.35 (17)	C13—C14—C15	125.30 (15)
C3—C2—H2	119.8	C13—C14—H14	117.3
C1—C2—H2	119.8	C15—C14—H14	117.3
C2—C3—C4	120.59 (17)	C23—C15—C16	117.06 (15)
C2—C3—H3	119.7	C23—C15—C14	123.01 (15)
C4—C3—H3	119.7	C16—C15—C14	119.90 (15)
C5—C4—C3	120.26 (17)	N2—C16—O2	118.93 (15)
C5—C4—H4	119.9	N2—C16—C15	125.40 (15)
C3—C4—H4	119.9	O2—C16—C15	115.64 (14)
C4—C5—C6	120.40 (17)	N2—C17—C18	118.43 (15)
C4—C5—H5	119.8	N2—C17—C22	122.48 (15)
C6—C5—H5	119.8	C18—C17—C22	119.09 (16)
C1—C6—C5	118.99 (16)	C19—C18—C17	120.17 (16)
C1—C6—C7	118.19 (15)	C19—C18—H18	119.9
C5—C6—C7	122.80 (16)	C17—C18—H18	119.9
C8—C7—C6	117.51 (16)	C18—C19—C20	119.84 (16)
C8—C7—C10	122.36 (16)	C18—C19—H19	120.1
C6—C7—C10	120.09 (15)	C20—C19—H19	120.1
C7—C8—C9	120.31 (15)	C21—C20—C19	121.85 (16)
C7—C8—C12	119.88 (16)	C21—C20—Cl1	120.09 (14)
C9—C8—C12	119.78 (15)	C19—C20—Cl1	118.05 (13)
N1—C9—C8	122.86 (16)	C20—C21—C22	118.66 (16)
N1—C9—C11	116.28 (16)	C20—C21—H21	120.7
C8—C9—C11	120.83 (16)	C22—C21—H21	120.7
C7—C10—H10A	109.5	C21—C22—C17	120.28 (15)
C7—C10—H10B	109.5	C21—C22—C23	122.08 (15)
H10A—C10—H10B	109.5	C17—C22—C23	117.62 (15)
C7—C10—H10C	109.5	C15—C23—C22	120.13 (15)
H10A—C10—H10C	109.5	C15—C23—H23	119.9
H10B—C10—H10C	109.5	C22—C23—H23	119.9
C9—C11—H11A	109.5	O2—C24—H24A	109.5
C9—C11—H11B	109.5	O2—C24—H24B	109.5
H11A—C11—H11B	109.5	H24A—C24—H24B	109.5
C9—C11—H11C	109.5	O2—C24—H24C	109.5
H11A—C11—H11C	109.5	H24A—C24—H24C	109.5
H11B—C11—H11C	109.5	H24B—C24—H24C	109.5
			
C9—N1—C1—C6	−1.2 (2)	C8—C12—C13—C14	2.2 (3)
C9—N1—C1—C2	177.38 (15)	C12—C13—C14—C15	−177.90 (16)
N1—C1—C2—C3	−179.95 (16)	C13—C14—C15—C23	35.7 (3)
C6—C1—C2—C3	−1.3 (2)	C13—C14—C15—C16	−146.59 (18)
C1—C2—C3—C4	−0.9 (3)	C17—N2—C16—O2	179.06 (14)
C2—C3—C4—C5	1.8 (3)	C17—N2—C16—C15	1.2 (2)
C3—C4—C5—C6	−0.6 (3)	C24—O2—C16—N2	3.1 (2)
N1—C1—C6—C5	−178.96 (16)	C24—O2—C16—C15	−178.80 (16)
C2—C1—C6—C5	2.5 (2)	C23—C15—C16—N2	−1.9 (2)
N1—C1—C6—C7	2.7 (2)	C14—C15—C16—N2	−179.71 (16)
C2—C1—C6—C7	−175.86 (15)	C23—C15—C16—O2	−179.79 (14)
C4—C5—C6—C1	−1.5 (3)	C14—C15—C16—O2	2.4 (2)
C4—C5—C6—C7	176.70 (17)	C16—N2—C17—C18	−178.75 (15)
C1—C6—C7—C8	−2.4 (2)	C16—N2—C17—C22	0.8 (2)
C5—C6—C7—C8	179.40 (16)	N2—C17—C18—C19	−177.67 (16)
C1—C6—C7—C10	175.34 (15)	C22—C17—C18—C19	2.7 (2)
C5—C6—C7—C10	−2.9 (2)	C17—C18—C19—C20	0.3 (3)
C6—C7—C8—C9	0.7 (2)	C18—C19—C20—C21	−2.3 (3)
C10—C7—C8—C9	−176.92 (16)	C18—C19—C20—Cl1	176.79 (14)
C6—C7—C8—C12	178.51 (14)	C19—C20—C21—C22	1.2 (3)
C10—C7—C8—C12	0.9 (2)	Cl1—C20—C21—C22	−177.88 (12)
C1—N1—C9—C8	−0.6 (2)	C20—C21—C22—C17	1.9 (2)
C1—N1—C9—C11	−178.50 (16)	C20—C21—C22—C23	−179.54 (15)
C7—C8—C9—N1	0.8 (3)	N2—C17—C22—C21	176.60 (15)
C12—C8—C9—N1	−176.98 (15)	C18—C17—C22—C21	−3.8 (2)
C7—C8—C9—C11	178.64 (17)	N2—C17—C22—C23	−2.0 (2)
C12—C8—C9—C11	0.8 (2)	C18—C17—C22—C23	177.52 (15)
C7—C8—C12—O1	−72.2 (2)	C16—C15—C23—C22	0.5 (2)
C9—C8—C12—O1	105.6 (2)	C14—C15—C23—C22	178.25 (15)
C7—C8—C12—C13	105.55 (19)	C21—C22—C23—C15	−177.31 (15)
C9—C8—C12—C13	−76.6 (2)	C17—C22—C23—C15	1.3 (2)
O1—C12—C13—C14	179.89 (18)		

### Hydrogen-bond geometry (Å, º)

**Table:**

*D*—H···*A*	*D*—H	H···*A*	*D*···*A*	*D*—H···*A*
C10—H10*B*···O1^i^	0.98	2.52	3.221 (2)	129

Symmetry code: (i) −*x*+3/2, *y*+1/2, −*z*+1/2.

## References

[bb1] Agilent (2013). *CrysAlis PRO* Agilent Technologies Inc., Santa Clara, CA, USA.

[bb2] Brandenburg, K. (2006). *DIAMOND* Crystal Impact GbR, Bonn, Germany.

[bb3] Farrugia, L. J. (2012). *J. Appl. Cryst.* **45**, 849–854.

[bb4] Joshi, R. S., Mandhane, P. G., Khan, W. & Gill, C. H. (2011). *J. Heterocycl. Chem.* **48**, 872–876.

[bb5] Prasath, R. & Bhavana, P. (2012). *Heteroatom Chem.* **2**3, 525–530.

[bb6] Prasath, R., Bhavana, P., Ng, S. W. & Tiekink, E. R. T. (2013*a*). *J. Organomet. Chem.* **726**, 62–70.

[bb7] Prasath, R., Sarveswari, S., Ng, S. W. & Tiekink, E. R. T. (2013*b*). *Acta Cryst.* E**69**, o1275. 10.1107/S1600536813019405 PMC3793770 24109357

[bb8] Sheldrick, G. M. (2008). *Acta Cryst.* A**64**, 112–122. 10.1107/S0108767307043930 18156677

[bb9] Westrip, S. P. (2010). *J. Appl. Cryst.* **43**, 920–925.

